# xploring Neuromelanin Accumulation in the Locus Corelulus: Implications for Early Alzheimer’s Disease Pathology

**DOI:** 10.1002/alz.092966

**Published:** 2025-01-03

**Authors:** Leslie Hassanein, David Weinshenker, Weinshenker Lab

**Affiliations:** ^1^ Emory University, Atlanta, GA USA; ^2^ Emory University School of Medicine, Atlanta, GA USA

## Abstract

**Background:**

Alzheimer’s Disease (AD) is a pressing global health concern, particularly among the elderly population. Early detection and intervention are vital for effective management. Recent research has identified the Locus Corelulus (LC) as one of the initial sites of pathology in AD, characterized by the degeneration of norepinephrine (NE) producing cells, resulting in cognitive and mood disturbances. This study investigates the relationship between neuromelanin (NM) and AD pathology, focusing on its accumulation in the LC. NM, a pigment‐like substance synthesized from catecholamine neurotransmitters such as NE, has been proposed to have a neuroprotective role. However, its potential toxicity remains unexplored.

**Method:**

We employed a novel approach involving the introduction of NM production in mice using a cre‐dependent human tyrosinase (hTyr) virus. This technique allowed us to observe significant NM accumulation in the LC and the emergence of neurodegenerative disease‐like phenotypes in mice. Our investigations extended to examining LC‐NE dependent behavior, NM accumulation, and cell death at distinct time points (1, 6, and 10 weeks).

**Result:**

Our results show a longitudinal degradation of the LC, with almost complete loss of the region at 6 weeks.

**Conclusion:**

The progressive LC loss and behavioral changes suggest NM’s involvement in AD pathogenesis. Further research is needed to explore NM‐induced neurodegeneration and its potential for early AD detection and intervention.

